# Risk factors for caries-free time: longitudinal study in early childhood

**DOI:** 10.11606/S1518-8787.2017051006558

**Published:** 2017-11-16

**Authors:** Maria Beatriz Barreto de Sousa Cabral, Eduardo Luiz Andrade Mota, Maria Cristina Teixeira Cangussu, Maria Isabel Pereira Vianna, Fabiana Raynal Floriano

**Affiliations:** I Universidade Federal da Bahia. Faculdade de Odontologia. Departamento de Odontologia Social. Salvador, BA, Brasil; II Universidade Federal da Bahia. Instituto de Saúde Coletiva. Salvador, BA, Brasil; III Ministério da Saúde. Departamento de Atenção à Saúde. Brasília, DF, Brasil

**Keywords:** Infant, Child, Preschool, Dental Caries, epidemiology, Risk Factors, Cohort Studies

## Abstract

**OBJECTIVE:**

To estimate time in days from the beginning of follow-up up to the development of dental caries in children under 30 months and to assess risk factors potentially affecting the development of the disease.

**METHODS:**

The study population of the cohort study were children attending public, private, and charitable day care centers in Salvador, Northeastern Brazil, followed up for fourteen months. We used the multivariate Cox regression to estimate risk and Kaplan-Meier method to estimate the caries-free time.

**RESULTS:**

Of the 495 children studied, 112 developed caries (22.6%). Mean caries-free time was 248.6 (SD = 96.2) days. The comparasion of curves by age group (> 24 months) and children attending public day care showed more caries in a shorter period (p < 0.00). The following variables were important risk factors for increased rate of caries: district of origin (HR = 1.88, 95%CI 1.27–2.77), category of day care (HR = 3.88, 95%CI 2.04–7.38), age (HR = 1.77, 95%CI 1.15–2.74), bottle-feeding before sleep time after the age of 12 months (HR = 1.62, 95%CI 1.04–2.51), presence of active white spots (HR = 2.70, 95%CI 1.07–6.80), and living in non-masonry house (HR = 1.68, 95%CI 1.02–2.76). The highest hazard ratio (HR = 4.60, 95%CI 2.80–7.42) was found for previous caries experience.

**CONCLUSIONS:**

Social variables were considered as of high risk for the development of dental caries.

## INTRODUCTION

Dental caries, a serious persistent public health problem issue, affects different groups from various socioeconomic backgrounds and predominantly specific age groups with severe presentations in early childhood that may lead to tooth loss^1^. A decline in the prevalence of caries in schoolchildren was seen in the last decade in Brazil after the implementation of collective actions, such as fluoridation of the public water system, topical use of fluoride in school programs, fluoride addition to toothpastes, and extended coverage of health services^2–4^.

However, caries remains a critical challenge in other age groups, such as younger children who have shown greatly morbidity, expressed by different prevalence rates^3–9^. Early caries in younger children have specific destructive characteristics and undesirable consequences, such as pain and infection. These manifestations can lead to inadequate nutritional patterns and consequently impair their physical development. The esthetic issue should also be emphasized, which may affect social interaction and result in social withdrawal and shyness^8,10–12^.

As any chronic cumulative disease, dental caries requires some time to be clinically evident. The early years of life are considered of greater risk for the development of dental caries. In addition, general and oral health conditions can be affected by factors that are present from birth, such as low schooling of the mother, low birth weight, and poor nutrition. This seems to increase the risk of dental caries, which is more prevalent in areas of greater social deprivation^12–15^. Antunes et al. have also suggested that social and biological risks, built up from childhood, subsequently have an impact on health during adult life. Furthermore, younger children with caries are also more likely to have this same condition in their permanent dentition^16^. Moreover, the likelihood of development of caries in children is related to the level of infection of their caretakers, frequency of exposure, immune status, and diet^10^ ^,13–17^.

Longitudinal studies, mostly developed in European countries, describe socioeconomic, demographic, and behavioral factors, clinical characteristics, and other characteristics related to the health of children as major risk factors for the development of caries. They point out the impact of socioeconomic factors, which are practically invariable for a limited time period^8^ ^,^ ^15^ ^,^ ^18^ ^,^ ^19^.

We intend to understand the process of oral health and disease from the theoretical background of social epidemiology. Life conditions are an expression of the resources available for the existence of social groups, determined by how these groups are included in the process of social reproduction under specific natural conditions and at a given point in time^20^ ^,^ ^21^. It is relevant to understand the concept of the way of life as a category comprising two dimensions: life conditions associated with environment conditions and resources required for life and determined by social consumption and life style, which in turn refers to socially and culturally established ways of living translated by habits^19–21^.

Therefore, epidemiological knowledge involves the search for an explanation and the change of health and disease conditions in their social dimension, by evidencing the effect of social inequalities in the health and disease processes, particularly during childhood. Since the Brazilian reality is strongly marked by health inequalities, among them dental caries, we emphasize the role epidemiology has as a key instrument for oral health surveillance. Following these principles and considering the limited epidemiological knowledge production in oral health in preschool children, as well as the scarcity of longitudinal studies, this study aimed to assess the association between the development of caries and socioeconomic and demographic conditions and behavioral characteristics, as well as the time span for caries development during the early years of life.

## METHODS

A prospective cohort study was carried out and we followed up children aged four to thirty months who attended public, private, or charitable day care centers in two health districts of the city of Salvador, Northeastern Brazil, between October 2002 and December 2003.

There was no sampling process. This was a census study with a convenience sample of all private, public, and charitable day cares identified in two health districts in the city of Salvador, State of Bahia. The Barra-Rio Vermelho district is located on the South of the city, involving neighborhoods of varying profiles of living conditions. Twenty day care centers were identified, being five of them public, eight private centers, and seven philantropic ones. The Cabula Beiru district is located on the North of the city, with a predominance of popular housing and many irregular communities, being an area with serious problems of urban infrastructure. In this district, we identified four public day care centers and seven philanthropic ones.

After contacting the day care, letters were sent to all those responsible for the children, explaining the research objectives, as well as the informed consent term to be signed. Of a total of 640 children enrolled, we obtained positive return from 86.9% of the guardians. The project was approved by the Research Ethics Committee of the Instituto de Saúde Coletiva of Universidade Federal da Bahia.

Data collection had two steps: socioeconomic and demographic data, oral health, and behavioral aspects obtained from a standardized interview with the mother or careegiver of each the child, and clinical examination. The considered variables were: age (≥ 24 months, < 24 months), sex (male, female), race (white, black or biracial), home district (Barra-Rio Vermelho, Cabula Beiru); type of day care (public, private, charitable); schooling of the mother or father (≥ high school or university, < high school or university); occupation of the mother or father (upper of average level, unemployed or low level in the Brazilian classification of occupations), age of the mother (≥ 20 years, < 20 years), family income *per capita* (from 0.38 to 7.75, 0.06 to 0.37 minimum wage); marital status of the mother (presence, absence of companion), number of siblings (one sibling, more than one sibling), number of persons perroom (two or less persons, more than two persons); frequency of drug use of the mother (no, yes: < once per week, more than once per week, including alcohol and illegal drugs), water in the residence (present, absent), mansonary house (yes, no), type of feeding (natural, artificial, mixed); time of bottle-feeding or artificial breastfeeding (≥ to 24 months, < 24 months), feeding during sleeptime (≥ 24 months, < 24 months), sucking during sleep (≥ 12 months, < 12 months), use of sugar (before six months, after six months), oral hygiene (before 12 months, after 12 months), frequency of brushing (twice a day or more, once a day or rarely, never). For the dichotomy of the variables, we used the median as the distribution parameter.

Clinical examination was conducted by a team consisting of an examiner and two notetakers with proper training. A proper pre-tested instrument was used. This included: number of present teeth, the presence of enamel hypoplasia, presence of visible plaque (VP), caries (dmft index), presence of active white spots (AWP), and trauma (partial or total loss of the tooth). The Kappa index for intra-examiner agreement ranged from 0.93 to 0.98 for each observed situation.

The children were examined on the same day care environment. Disposable gloves, wooden spatulas, and a white light lantern were used. The considered diagnostic criteria were: the presence of plaque, i.e., visible buildup of soft deposits, loose or stuck on the buccal surfaces of the upper deciduous incisors, identified during visual inspection. We emphasize that this condition was evaluated only in the second examination by the same dentist in an average interval of six months after the first one; therefore, we have a loss of sixty-one cases. The following definitions were considered in this study: active white spots (AWP): when the enamel surface presented opaque white color and irregular appearance, active caries lesions (AC): clinical evidence of caries activity, inactive caries (IC): chronic aspect of cavitation and dark coloring, dental filling (DF): presence of definitive restorative material, and extraction indicated (EI): presence of extensive coronary destruction and consequent pulp involvement. The child was defined with caries if one or more cavitations were present with acute or chronic aspect. The AWP were analyzed as a risk variable, since they have a reversible nature. The final examinatation was the criteria to determine wheter the child was free from caries or not for the whole period.

A survival analysis^22^ approach was used to estimate the likelihood of children to be caries-free for a certain period and to assess the relative significance of its associated factors. A descriptive analysis was performed to describe the frequency distribution and mean time by the selected variables. For the survival analysis, the dependent variable was caries-free time, in days, defined as the difference between the date of diagnosis during follow-up and the date of the first examination.

Failure was defined as caries development, diagnosed by the presence of a cavity. For right-censored data, we considered the end of follow-up at a present date or lost to follow-up. For left-censored data, we considered the start date of follow-up for each child in the study. The selection of the independent variables was based on the information available in the literature and the findings obtained in a previous stratified analysis of the association between exposure factors and dental caries.

Univariate analysis of the time span for the development of caries for each study variable was conducted using Kaplan-Meier^23^ procedure to describe survival functions by applying log-rank test to acess the likelihood between these functions by category, equivalent to the chi-square test with one degree of freedom. For those variables with a statistically significant difference between survival functions, proportionality was estimated by graphically comparing -*log[-log]* curves and they were included in the Cox regression analysis.

A comparison between the hazard function values from the development of caries by independent variable was carried out by Cox regression analysis using Breslow method^23^. The multivariate analysis was performed only with variables with p < 0.20 in the univariate step. After this, all the variables that had p < 0.20 were considered together. In the final model, for those that had p < 0.05 in the multivariate analysis, we estimated the risk ratio or hazard ratio (HR). Finally, we carried out Cox- Snell residual analysis to estimate the adjustment of the model^3^.

Hazard ratios were obtained in the univariate and multivariate analyses of regression models, and 95% confidence intervals were estimated using Greenwood method. Results were regarded as statistically significant if they matched the likelihood of chance (p) equal to or below the 5% level. Three full models were created. The first model included all the children who did not have any caries in the beginning of the study (n = 455). The second one included the children who did not have any caries in the beginning of the study attending public day cares (n = 305). The third one included all children studied (n = 495) considering their previous caries experience and, in this case, caries increment over the study period.

In each model, the following exposure factors were grouped: age, race, district of origin, type of day care, average family income *per capita* (in minimum wages), schooling of the mother, schooling of the father, occupation of the father and mother, living or not in a masonry house, duration of bottle-feeding, bottle-feeding before and during sleep time, use of sugar in the diet of the children, presence of bacterial plaque and active white spots, variables associated with oral hygiene habits (frequency of brushing, use of toothbrush and toothpaste), and frequent use of drugs. In the third model, in addition to these variables, we included previous caries experience.

From the first full model, after excluding the variables that were not statistically significant and applying the maximum likelihood estimation method for the goodness of fit test of the reduced models, the best final explanatory model was defined showing adjusted hazard ratios, 95% confidence intervals, and p-values equivalent to the application of the z-test for regression coefficients. Analyses were performed using the Stata software program, version 7.0^24^.

## RESULTS

**Figure 1:**
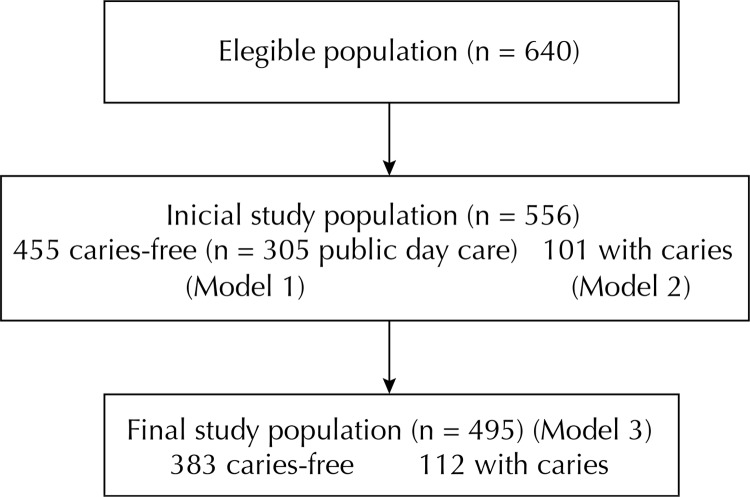
Flowchart of the study population.

A total of 495 children were followed up, being 214 (43.2%) aged between 13 and 24 months and 237 (47.9%) females. Approximately 112 children developed caries and they were evident early in the course of follow-up, as shown in Figure 1 by the increased rate of decline in the survival curve in the beginning of the study. Mean time for the development of caries was 248.6 days (standard deviation [SD] = 96.2 days), ranging between 89 and 411 days.

**Figure 2:**
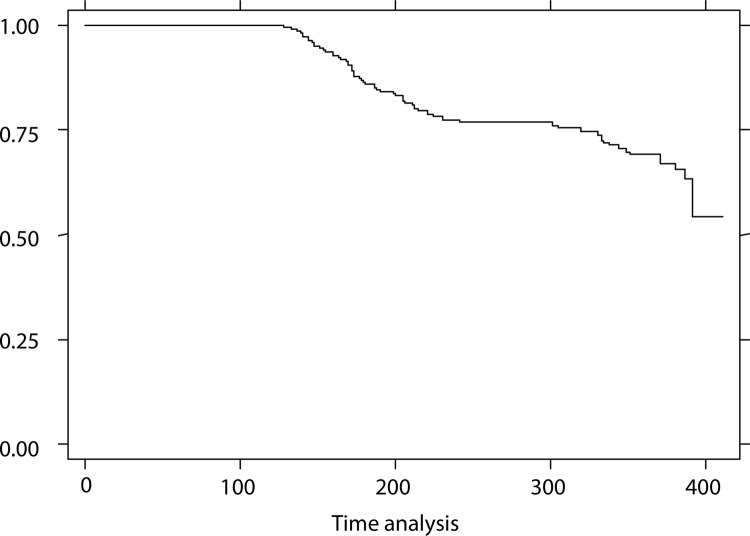
Kaplan-Meier survival function curve based on dental caries-free time for children attending public, private, and charitable day care centers in the municipality of Salvador, Brazil, 2002–2003.

**Figure 3:**
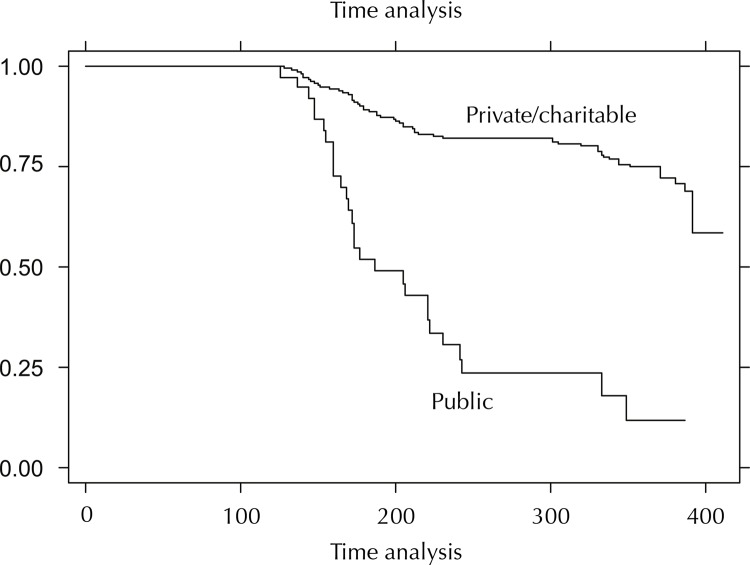
Kaplan-Meier survival function curve based on dental caries-free time by category of day care for children attending public, private, and charitable day care centers in the municipality of Salvador, Brazil, 2002–2003.

**Figure 4:**
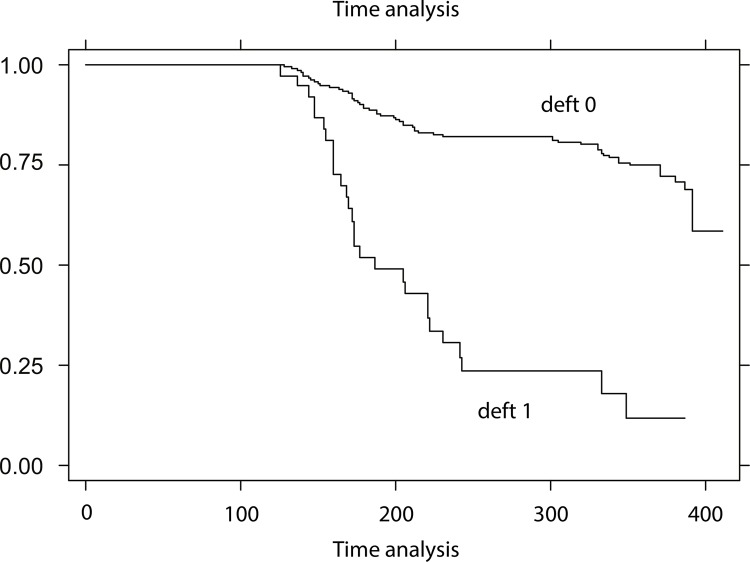
Kaplan-Meier survival function curve based on dental caries-free time by previous experience of caries for children attending public, private, and charitable day care centers in the municipality of Salvador, Brazil, 2002–2003.

The comparison of the survival curve of children with no caries, by age group, showed statistically significant difference (p < 0.0001); children older than 24 months and those attending public day cares had more caries in a shorter time period (Figure 2). The comparison of the survival curve of children with no caries by their previous caries experience showed a statistically significant difference (p < 0.001); those with previous caries experience developed new caries in a shorter period (Figures 3 and 4).

Kaplan-Meier survival estimate

Kaplan-Meier survival estimates by day care

Deft = 0 (caries-free at baseline) and deft = 1 (dental caries at baseline)

The assessment of the exposure factor associated with the time span from the beginning of follow-up to the development of caries showed that caries-free time was shorter for children from the Cabula-Beiru health district, who attended public day care, aged more than 24 months, black or biracial, with mothers or fathers with low schooling who were either unemployed or had low-skilled jobs and thus had lower family income, who were often bottle fed before bedtime over 24 months of age or bottle fed during sleep time over 12 months of age, with sugar in their diet, with frequent use of drugs, who had bacterial plaques or active white spots, who had previous experience of caries, and who lived in non-masonry houses (Table 1). Other variables were not significant in the prediction and were not included in the results.

**Table 1:** Number of children followed up, time under risk of developing dental caries, comparison of expected and observed caries cases, and hazard ratio according to exposure factors in children attending public, private, and charitable day care centers in Salvador, State of Bahia, Brazil, 2002–2003.

Exposure factors	n	Time under risk (days)	Observed Events (n)	Expected events (n)	p^a^	Hazard ratio^b^	95%CI
Age							
≤ 24 months	277	69.01	42	62.01		1,0	
> 24 months	218	54.05	70	49.92	0.000	2.07	1.41–3.03
Race							
White	64	15.85	3	13.84		1.0	
Black/Biracial	431	107.22	109	98.22	0.005	5.11	1.62–16.12
District of origin							
Barra-Rio Vermelho	305	78.99	58	74.71		1.0	
Cabula-Beiru	190	44.07	54	37.33	0.001	1.93	1.31–2.84
Category of day care							
Private/Charitable	166	41.86	22	38.72		1.0	
Public	329	81.21	90	73.34	0.001	2.17	1.36–3.47
Schooling of the mother^c^							
≥ High school/University	254	64.04	42	60.71		1.0	
< High school/University	236	57.57	70	51.22	0.000	1.98	1.35–2.91
Schooling of the father^c^							
≥ High school/University	205	52.59	29	47.13		1.0	
< High school/University	246	59.43	68	49.93	0.000	2.23	1.44–3.45
Occupation of the mother^c^							
Upper or average level CBO	82	21.61	4	20.35		1.0	
Low CBO or unemployed	410	100.811	108	91.77	0.000	6.04	2.22–16.4
Occupation of the father^c^							
Upper or average level CBO	99	26.00	9	25.39		1.0	
Low CBO or unemployed	335	81.14	91	74.75	0.000	3.49	1.75–6.95
Family income (minimum wage)							
≤ 0.38–7.75	179	46.36	29	44.42		1.0	
< 0.06–0.37	290	70.46	81	65.56	0.003	1.89	1.24–2.90
Masonry house							
Yes	338	100.14	80	92.23		1.0	
No	187	22.92	32	19.87	0.003	1.87	1.24–2.84
Bottle-feeding							
≤ 24 months	426	105.37	85	95.32		1.0	
> 24 months	69	17.69	27	16.74	0.007	1.81	1.17–2.79
Bottle-feeding before bedtime							
≤ 24 months	325	80.85	57	73.11		1.0	
> 24 months	170	42.21	55	38.92	0.002	1.81	1.25–2.62
Bottle-feeding during sleep time							
≤ 12 months	267	65.73	46	59.64		1.0	
> 12 months	228	57.34	66	52.43	0.011	1.63	1.12–2.38
Use of sugar							
No	60	14.52	4	12.76		1.0	
Yes	435	108.54	108	99.34	0.015	3.46	1.27–9.38
Active white spots							
No	468	117.32	98	107.57		1.0	
Yes	27	5.74	14	4.51	0.000	3.45	1.96–6.07
Frequent use of drugs							
No	334	94.89	80	89.31		1.0	
Yes	204	28.17	32	22.62	0.028	1.59	1.05–2.41
Presence of visible plaque							
No	103	27.01	6	25.53		1.0	
Yes	392	96.05	106	86.51	0.000	5.32	2.33–12.13
Previous experience of caries							
No	455	114.91	84	105.70		1.0	
Yes	40	8.15	28	6.30	0.000	5.81	3.75–8.99

CBO: Brazilian classification of occupations

^a^ p-value equivalent to log-rank test for the survival function of likelihood – chi-square test with 1 degree of freedom.

^b^ Comparison of hazard functions by caries obtained in the univariate Cox regression analysis using Breslow method. Failure was defined as caries development.

^c^ Missing data.

Variables related to oral hygiene practices (higher frequency of brushing, use of toothbrush and toothpaste) were not included. The hazard ratios of these variables were not statistically significant. No statistically significant association was found between survival functions and the sex of the children and other variables related to prenatal history and other health conditions (data not shown).

When all children with no caries in the beginning of the study were considered, the multivariate analysis showed that disease-free time was shorter for those from the Cabula-Beiru health district, attending public day care, aged more than 24 months, bottle fed during sleep time over 12 months of age, with active white spots, and who lived in non-masonry houses. Then, when public day care children were considered, Cox model showed that disease-free time was shorter for children with similar characteristics as described above for all children (Table 2).

**Table 2:** Hazard ratios adjusted for dental caries by exposure factors obtained in the multivariate Cox regression analysis in children under 30 months. Salvador, State of Bahia, Brazil, 2002–2003.

Exposure factors	HR*	95%CI	p
Model 1			
Cabula-Beiru district	2.04	1.27–3.26	0.003
Public day care	3.88	2.04–7.38	< 0.001
Age > 24 months	1.77	1.15–2.74	0.010
Active white spots	2.70	1.07–6.80	0.035
Bottle-feeding during sleep time after 12 months of age	1.62	1.04–2.51	0.032
Non-masonry house	1.68	1.02–2.76	0.040
Model 2			
Cabula-Beiru district	1.88	1.13–3.13	0.015
Age > 24 months	1.83	1.15–2.92	0.010
Active white spots	2.96	1.05–8.31	0.039
Bottle-feeding during sleep time after 12 months of age	1.65	1.03–2.66	0.038
Non-masonry house	2.06	1.22–3.47	0.006
Model 3			
Cabula-Beiru district	1.88	1.27–2.77	0.002
Public day care	2.80	1.73–4.51	< 0.001
Age > 24 months	1.70	1.14–2.55	0.010
Active white spots	1.88	1.01–3.49	0.045
Previous experience of caries	4.60	2.80–7.42	< 0.001

Model 1: It includes all children attending private, public, and charitable day care with no caries in the beginning of the study (n = 455).

Model 2: It includes all children attending public day care with no caries in the beginning of the study (n = 305).

Model 3: It includes all children with previous experience of caries in the beginning of the study (n = 495).

* Hazard ratios obtained in the Cox regression analysis using Breslow method. Failure was defined as caries development. The degree of goodness of the model was tested using the maximum likelihood method.

In the third model, after including the variable previous experience of caries, the factors that mostly shortened disease-free time for all exposed children were: being from the Cabula-Beiru health district, attending public day care, being aged more than 24 months, having incipient lesions (active white spots), greater hazard ratio (HR = 4.60, 95%CI 2.80–7.42), and previous caries experience (Table 2).

## DISCUSSION

As the objective of this study was to increase the understanding of the process of health and caries disease during early childhood in an urban area characterized by pronounced social inequalities, we chose the survival analysis to assess the association between the development of caries and socioeconomic, demographic, and behavioral factors in relation to the dependent variable time. In this study, 112 children developed caries in a mean time of 248 days. The comparasion of survival curve by age, public day care, and previous caries experience showed a development of caries in a shorter time period (p < 0.00).

To identify the individuals susceptible to caries who comprise the so-called polarization group and to provide them with individual care, we suggest high-risk strategies^25–27^, which could have a better cost-benefit from the application of the resources available.

The available knowledge has shown that caries in early childhood is associated with several factors, including oral hygiene habits, low birth weight, *S. mutans* infection, hypomineralization, fluoride exposure, intake of saccharose-rich food, among others, making it a multifactorial disease^8,11,14–19,^ ^25^ ^,^ ^28^ ^,^ ^30^. Therefore, studies investigating caries have to address increasingly complex challenges, particularly longitudinal studies, since different exposures can vary over time. Besides the individual clinical and behavioral aspects, we highlight the role of socioeconomic and demographic factors that significantly contribute to shorten the time span for the development of caries.

In the analysis of time for the development of caries in children attending public, private, and charitable day care, we estimated an average time of 211 days, that is, children remain disease-free for seven months. To assess major exposure factors potentially affecting this time, we noted the following aspects in both the model including all children and the one including only public day care children: district of origin and category of day care attended, age, presence of active white spots, extended bottle-feeding during sleep time, and living in a non-masonry house.

According to the literature^8^ ^,^ ^28^, the rate of development of dental caries in deciduous teeth is higher than in permanent teeth, as evidenced in this study population: the original deft index triplicated in one year. However, we could note a polarization or high disease activity in the group of children who present caries in its most active and severe form; thus, this group has a higher risk of developing caries. Consequently, the time required for the development of caries in this group is shorter as multiple risk factors interact to produce this outcome. Among these multiple risk factors, the district of origin and mainly the category of day care could be considered as proxy variables of social conditions as they highly distinguish the profile of these children: children attending public day care have poorer life conditions, for instance those living in non-masonry houses. Accordingly, this is also reflected in their oral health and especially in the time that their health status is affected. Therefore, public day cares shorten the time for the development of caries when compared to private day cares. No caries was detected in children of private day cares during the study follow-up.

Regarding the age of thechildren, the older the child, the higher the risk of caries, as children are more exposed to various determinants and to additional tooth eruption. Corroborating the literature that highlights the relevance of the relationship between early pattern of caries and its incidence^25^, we verified in the literature that children older than 24 months are prone to develop caries in a relatively short time from the synergistic action of multiple risk factors^3^ ^,^ ^11^ ^,^ ^26^ ^,^ ^28^ ^,^ ^29^.

The impact of behavioral factors should be highlighted again, especially those related to eating habits. Extended bottle-feeding during sleep time in children older than two years shortens the time for the development of caries from the action of the sugar found in milk or baby formulas, the higher plaque formation on dental surfaces, and reduced saliva flow while children are asleep^7–9,15,16,29^.

We showed that oral health conditions in the beginning of follow-up, such as visible plaque, considerably contribute to shorten the time for the development of caries^14^ ^,^ ^15^ ^,^ ^30^. Consequently, these cavities are a major factor for increasing the rate for developing new caries, which was confirmed in the analysis of the model including previous experience of caries. The major exposure factors potentially affecting the time for the development of caries were as follows: district of origin, category of day care, age, presence of active white spots, and, with higher risk ratio, having caries in the beginning of the study. In other words, the presence of caries is a condition for developing new caries^8^ ^,^ ^11^ ^,^ ^14^ ^,^ ^16^ ^,^ ^18^ ^,^ ^19^ ^,^ ^28^ ^,^ ^30^. This is relevant not only for the development of programs to “reestablish” lost health but also for the implementation of actions prioritizing health prevention and maintenance.

In the final model, after adjusting for risk factors shortening caries-free time, we were left with behavioral factors related to life style, such as extended bottle-feeding during sleep time, as well as distal, external factors related to the life conditions of the population studied, such as type of housing.

To ensure the control of caries as early as possible and to minimize treatment costs, since adequate restorative therapy is seemingly not provided to those children with high disease activity before the age of three, the current oral health model should undergo appropriate changes. Adequate technical and financial resources and trained providers need to be allocated for preventive and control actions. As risk factors such as lower schooling of the mother, low birth weight, and poor nutrition, seem to increase dental caries risk^14^ ^,^ ^15^ ^,^ ^19^ ^,^ ^28^ ^,^ ^30^, different intervention strategies need to be used^20,^ ^21^.

Therefore, education actions need to be increased mainly focusing on the promotion of positive changes in eating habits and oral hygiene as early as possible, confronting deeply rooted behavioral and cultural habits. Additionally, a population-based approach should be taken aimed at controlling disease determinants, using massive strategies to control and change social behaviors. As for oral health care, fluoridation of the public water system is legitimized as one of the main measures to reduce caries prevalence in children aged up to 12 years^2^. From a comprehensive children’s health care, behavioral changes are also needed, promoting maternal breastfeeding and balanced diets, as well as actions with a positive impact on general and oral health in the day care settings.

Intermediate- and long-term strategies can contribute to improve current oral health and move the epidemiological transition period to a new age, when individuals will actually have good oral health and will also be able to maintain it.

We reinforce the role of socioeconomic and demographic factors as major contributors to shortening the time for the development of caries. Bringing to light again the issue of social and, consequently, health inequalities, actions to reduce them will be only effective in the daily life of persons after profound transformations in economic production relations take place resulting in better life conditions^12^ ^,^ ^15^ ^,^ ^20^ ^,^ ^25^.

A limitation of this study was the use of the tooth unit for the measurement of case incidence. Potentially, the surface unit could bring greater accuracy to that extent, but it was not investigated in this study. A possible misclassification may have happened from the use of questionnaires or the criteria adopted in the variables used.

Moreover, epidemiological knowlegde will be a valuable technical and political instrument to link together various strategies and allow the envisioning of new ways of thinking and approaching health issues, especially in populations with greater need of care, to achieve more adequate health indicators.
